# Particulate Matter and Health Risk under a Changing Climate: Assessment for Portugal

**DOI:** 10.1100/2012/409546

**Published:** 2012-05-01

**Authors:** Daniela Dias, Oxana Tchepel, Anabela Carvalho, Ana Isabel Miranda, Carlos Borrego

**Affiliations:** Centre for Environmental and Marine Studies and Department of Environment and Planning, University of Aveiro, 3810-193 Aveiro, Portugal

## Abstract

The potential impacts of climate-induced changes in air pollution levels and its impacts on population health were investigated. The IPCC scenario (SRES A2) was used to analyse the effects of climate on future PM10 concentrations over Portugal and their impact on short-term population exposure and mortality. The air quality modelling system has been applied with high spatial resolution looking on climate changes at regional scale. To quantify health impacts related to air pollution changes, the WHO methodology for health impact assessment was implemented. The results point to 8% increase of premature mortality attributed to future PM10 levels in Portugal. The pollution episodes with daily average PM10 concentration above the current legislated value (50 **μ**g*·*m^−3^) would be responsible for 81% of attributable cases. The absolute number of deaths attributable to PM10 under future climate emphasizes the importance of indirect effects of climate change on human health.

## 1. Introduction

Climate change affects human health by a combination of direct and indirect processes. Thus, the abrupt change of temperatures leading to heat waves or cold spells has become widespread, causing fatal illnesses, such as heat stress or hypothermia, as well as increasing death rates from heart and respiratory diseases. According to the World Health Organization (WHO), the statistics on mortality and hospital admissions show that death rates increase during extremely hot days, particularly among very old and very young people living in cities. In Portugal, during the European heat wave of 2003, a total of 2399 excessive deaths were estimated which implied an increase of 58% over the expected deaths [1].

The indirect effects of climate change on human health are related, among others, to the changes in air pollution levels under future climate. Thus, changes in the temperature, humidity, wind, and precipitation that may accompany future climate can deeply impact air quality because of induced changes in the transport, dispersion, and transformation of air pollutants at multiple scales [2, 3]. According to Sheffield et al. [4], climate change could cause an increase in regional summer ozone-related asthma emergency department visits for children aged 0–17 years of 7.3% across the New York metropolitan region by the 2020s. When population growth is included, the projections of morbidity related to ozone were even larger. The authors also highlighted that the use of regional climate and atmospheric chemistry models makes possible the projection of local climate change health effects for specific age groups and specific disease outcomes.

The potential impact of climate change on particulate matter (PM) is of major concern because their concentrations are most likely to increase under a changing climate [5–7] and because future changes in particulate matter concentrations are likely the most important component of changes in mortalities attributable to air pollution in future scenarios [8]. Over the last few decades, human exposure to particulate air pollution has been associated with human mortality and morbidity, as well as a broad range of negative health outcomes at levels usually experienced by populations due to short- and long-term exposure to particulate matter [9–14]. The European directive (2008/50/CE) revised the limit values for PM10 (particulate matter with an aerodynamic diameter less than or equivalent to 10 *μ*m) previously defined by the Framework Directive (1999/30/EC) and set up new quantitative standards for PM2.5 (particulate matter with an aerodynamic diameter less than or equivalent to 2.5 *μ*m). Nevertheless, PM threshold levels to which exposure does not lead to adverse effects on human health have not yet been identified and given that there is a substantial inter-individual variability in exposure and in the response, it is unlikely that any standard or guideline value will lead to a complete protection for every individual against all possible adverse health effects of particulate matter [15].

For Portugal, studies show frequent exceedances of EU directive targets for air quality [16]. WHO has recently identified that Portugal is one of the 80 countries that exceed the reference values for particulate matter [17]. In addition, particulate emissions decreased in most European countries between 1990 and 2008 except for Portugal, Bulgaria, Romania, Malta, Finland, Denmark, Latvia, and Spain, where increases were recorded [18]. However, studies focusing on the health impacts of air quality in Portugal are very few. Several studies concerning the impact of meteorological factors on human health and the first attempt to relate air pollution levels and morbidity for Portugal have been published [1, 19–22]. The authors [20] highlight that under future climate the meteorological conditions will be more favourable for high ozone levels (low wind speed and high temperature) that could lead to impacts on human health. Recently, a number of studies on quantitative impact assessment of air pollution on mortality in Portuguese cities have emerged [23, 24] providing information on the association of current pollution levels with adverse health effects.

The main aim of the current study is to quantify the potential impact of short-term exposure to PM10 on population health under future climate. For this purpose, climate change scenario simulated with high temporal and spatial resolution is combined with health impact assessment (HIA). Air pollution modelling for the future scenario is performed assuming no changes in the PM10 precursor emissions in comparison with the reference situation thus allowing quantification of the climate change effect independently from the other factors that affect the pollution levels. The present study provides quantitative information on forecast of the health impact attributable to air pollution under a changing climate relevant for climate change mitigation and health policies.

## 2. Material and Methods

The potential impact on climate-induced human health effects caused by changes in PM10 concentrations over the continental Portugal is investigated using combined atmospheric and impact assessment modelling. The study is implemented in two main steps: (i) numerical simulation of PM10 concentrations over Portugal under the IPCC SRES A2 scenario and (ii) estimation of the number of deaths attributable to the changes in PM10 levels in the atmosphere under climate change.

**Figure 1:**
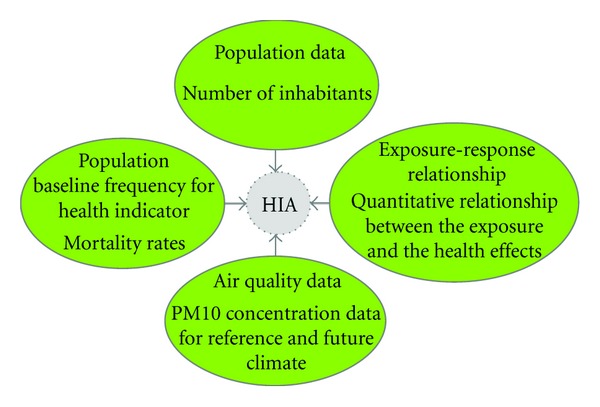
Schematic representation of the input information required by the health impact assessment performed in this study.

To quantify the health impact related with air pollution changes, the WHO methodology [25] was adapted and applied to the study area using the input information schematically presented in Figure 1.

### 2.1. Air Quality Modelling under Climate Change

**Figure 2:**
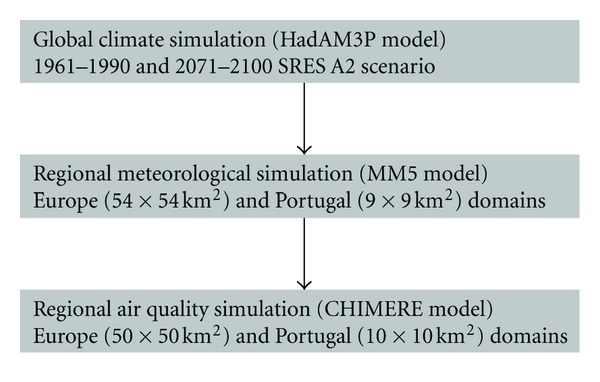
Schematic representation of the air quality numerical simulation.

The air quality modelling was performed for a reference and a future climate scenarios first at the European scale and then over Portugal [26]. For this purpose, global climate simulations provided by the HadAM3P model were used to drive the air quality modelling system as represented in Figure 2. The climate conditions for 1961–1990 are considered to characterize the reference situation, and predictions for 2071–2100 are used for the future climate in accordance with the IPCC SRES A2 scenario [27]. This scenario is considered to be the highest emission scenario and the carbon dioxide (CO_2_) concentrations reaching 850 ppm by 2100. In this sense, we are assessing the worst scenario with regard to air quality changes.

The air quality modelling system is based on the chemistry transport model CHIMERE [28, 29] forced by the mesoscale meteorological model MM5 [30]. The MM5/CHIMERE modelling system has been widely applied and validated in several air quality studies over Portugal [31–33] showing performance skills within the range found in several model evaluation studies using different air quality models [34, 35]. The MM5/CHIMERE modelling system has already been used in several studies that investigated the impacts of climate change on air pollutants levels over Europe [36] and specifically over Portugal [26]. The MM5 mesoscale model is a nonhydrostatic, vertical sigma coordinate model designed to simulate mesoscale atmospheric circulations. The selected MM5 physical options were based on the already performed validation and sensitivity studies over Portugal [37] and over the Iberian Peninsula [38]. A detailed description of the selected simulation characteristics is presented in [26]. The MM5 model generates the several meteorological fields required by the CHIMERE model, such as wind, temperature, water vapour mixing ratio, cloud liquid water content, 2 m temperature, surface heat and moisture fluxes, and precipitation.

CHIMERE is a tri-dimensional chemistry-transport model, based on the integration of the continuity equation for the concentrations of several chemical species in each cell of a given grid. It was developed for simulating gas-phase chemistry [28], aerosol formation, transport, and deposition [29, 39] at regional and urban scales. CHIMERE simulates the concentration of 44 gaseous species and 6 aerosol chemical compounds. In addition to the meteorological input, the CHIMERE model needs boundary and initial conditions, anthropogenic emission data, and the land use and topography characterization. The modelling system was firstly applied at the European scale (with 50 × 50 km^2^ resolution) and then over Portugal using the same physics and a simple one-way nesting technique, with 10 × 10 km^2^ horizontal resolution. The European domain covers an area from 14 W to 25 E and 35 N to 58 N. Over Portugal, the simulation domain goes from 9.5 W to 6 W and 37 N to 42.5 N [26]. The vertical resolution of CHIMERE model consists of eight vertical layers of various thicknesses extending from ground to 500 hPa. Lateral and top boundaries for the large-scale run were obtained from the LMDz-INCA (gas species) [40] and GOCART (aerosols) [41] global chemistry-transport models, both monthly mean values. The same boundaries conditions were used for both scenarios, since the objective is to only change the meteorological driver forcing. For the Portugal domain, boundary conditions are provided by the large-scale European simulation.

The CHIMERE model requires hourly spatially resolved emissions for the main anthropogenic gas and aerosol species. For the simulation over Europe, the anthropogenic emissions for nitrogen oxides (NO_x_), carbon monoxide (CO), sulphur dioxide (SO_2_), nonmethane volatile organic components (NMVOC) and ammonia (NH_3_) gas-phase species, and for PM2.5 and PM10 are provided by EMEP (Co-operative Programme for Monitoring and Evaluation of the Long-range Transmission of Air Pollutants in Europe) [42] with a spatial resolution of 50 km. The national inventory INERPA was used over the Portugal domain [32].

Reference and the IPCC SRES-A2 climate scenario over Europe and over Portugal were simulated by dynamical downscaling using the outputs of HadAM3P [43], as initial and boundary conditions to the MM5 model. The MM5 model requires initial and time-evolving boundary conditions for wind components, temperature, geopotential height, relative humidity, surface pressure, and also the specification of SSTs. Carvalho et al. [26] discuss the global model HadAM3P and the MM5 ability to simulate the present climate. The HadAM3P was selected to drive the MM5 model because a previous work [44] has already concluded that the HadAM3P accurately reproduces the large-scale patterns, namely, the 500 hPa fields. The 500 hPa height reflects a broad range of meteorological influences on air quality. The authors concluded that the HadAM3P is able to capture the mean patterns of the circulation weather types. The obtained results give confidence to use the HadAM3P outputs as initial and boundary conditions for regional simulations.

To evaluate the influence of climate change on air quality, the anthropogenic emissions were kept constant (to the year 2003) in the simulations for the future climate and were not scaled in accordance with the IPCC SRES A2 scenario. This idealized regional model simulation provides insight into the contribution of possible future climate changes on the 3D distribution of particulate matter concentrations. The MM5/CHIMERE simulations were conducted from May 1st to October 30th for the reference year (1990) and for the future scenario year (2100). Both simulations had the same chemical boundary conditions. Following this methodology, it is possible to analyse the changes caused by climate change only. In Carvalho et al. [26], a detailed analysis of the MM5/CHIMERE modelling system application under climate change has been presented and validated.

### 2.2. Population Analysis

**Figure 3:**
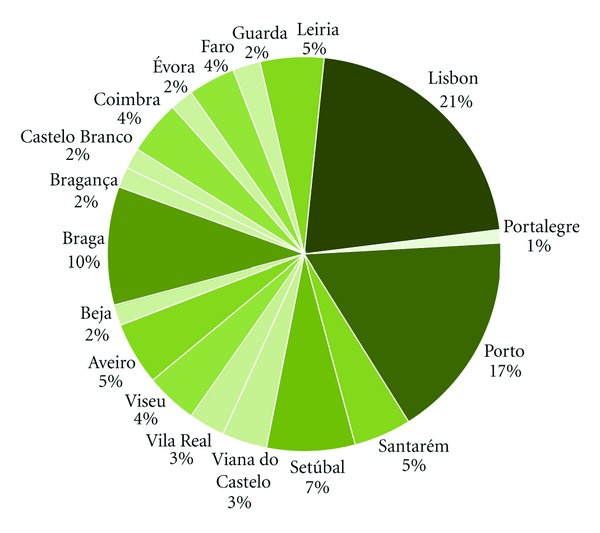
Distribution of demographic data by district in 2001.

Population size, composition, and health status were analysed for the study area as important elements required for the health impact assessment. According to National Institute of Statistics, the resident population in Portugal in 2001 was 9,869,343 inhabitants [45]. Lisbon and Porto are emphasized as the most densely populated agglomerations representing about 38% of total national population (Figure 3).

**Figure 4:**
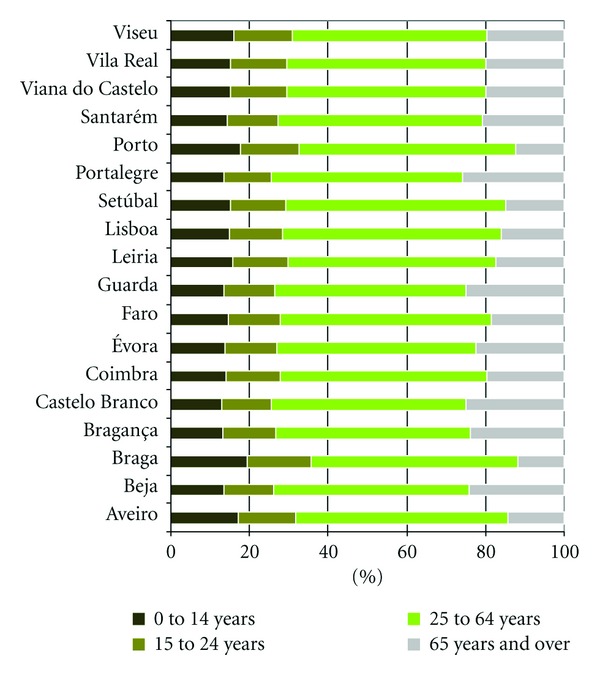
Distribution of population by age group for each Portuguese district in 2001.

The distribution of population by age groups is presented in Figure 4 stressing different proportion between active and older population for each district.

**Figure 5:**
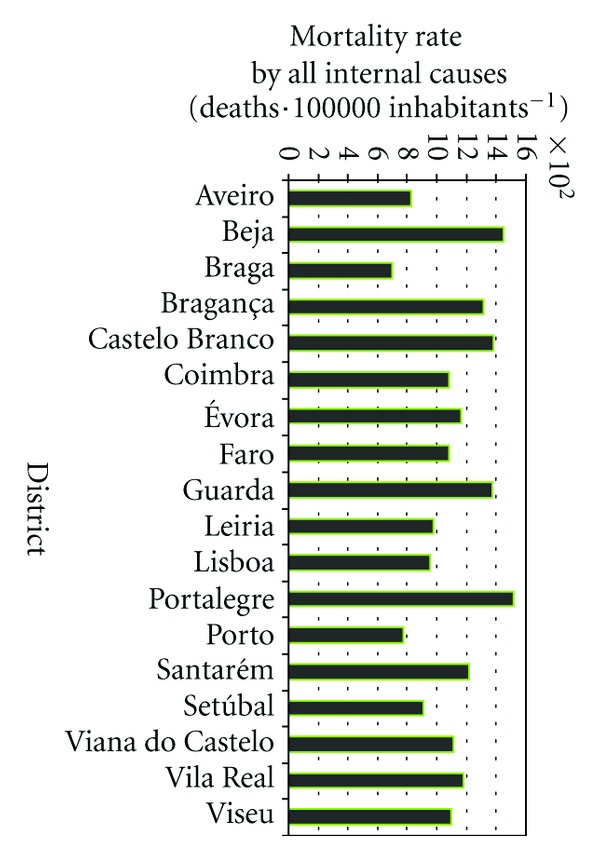
Annual mortality rate by all internal causes for each Portuguese district (deaths·100000 inhabitants^−1^) [46].

The health indicator considered in this study includes all-cause mortality (except external causes) (ICD-10 codes A00-R99) expressed as daily mortality rates in the number of deaths per 100000 inhabitants.  Figure 5 presents the distribution of annual mortality rate by district based on DGS [46].

**Figure 6:**
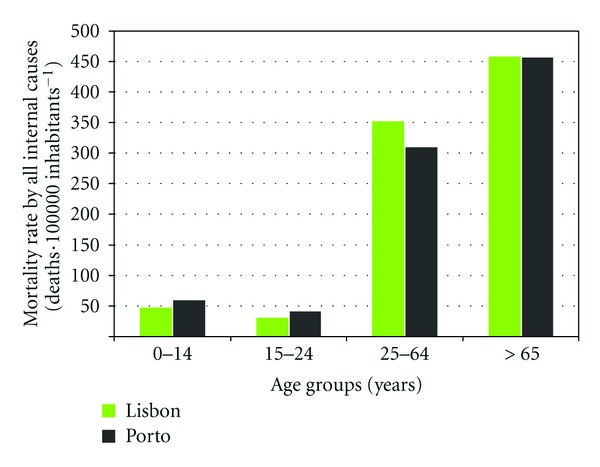
Annual mortality rate by all internal causes in Lisbon and Porto districts by age groups.

As could be seen, there is not a homogeneous distribution of mortality rate by the districts in Portugal. In general, the highest mortality rate by all internal causes is observed for the regions with higher proportion of older population as presented previously in Figure 4. Although, the Lisbon district indicates greater mortality rate than Porto with main difference in the mortality rate for age group 25–64 years (Figure 6).

### 2.3. Health Impact Assessment

A methodology to quantify health effects is conducted in terms of number of cases attributable to air pollution that may be prevented by reducing current levels of PM10 [25, 47]. An estimate of attributable deaths (AD) is obtained from the average number of deaths ($\overset{®}{y}$), the regression coefficient *β* provided by epidemiology-based exposure-response functions, and the difference between the daily average concentration ($\overset{®}{x}$) and a reference value under a given scenario (x*):

(1) $$\text{AD}=\overset{®}{y}\times \beta \left(\overset{®}{x}-x^{*}\right).$$

The EIS-PA model, developed by French Surveillance System on Air Pollution and Health as a support tool for automated and standardized health risk assessment [48], is used in this study to calculate the number of premature deaths prevented annually due to the reduction of PM to the selected “target” concentration. The results of EIS-PA model application provide estimates of the health outcomes related to short-term (1 or 2 days) exposure.

The exposure-response function, expressed as Relative Risk (RR) per 10 *μ*g·m^−3^, from epidemiological studies recommended by the European study [47] was adopted, considering the Relative Risk (RR) of 1.006 (95% CI (1.004–1.008)) for all-cause mortality (except external causes) to assess the effects on human health associated with the very short-term PM10 exposure (1 or 2 days) [49].

The time series of PM10 concentrations for future climate scenario together with demographic data and specific health indicators were considered in accordance with the Apheis guidelines [47] and used as input in the EIS-PA model [48]. The health impact assessment is implemented for two air pollution scenarios: (i) a simulation for current climate (year 1990) and projected 2100 PM10 levels under the IPCC SRES A2 scenario; (ii) for the air pollution reduction scenario considering the legislation limit values of daily average 50 *μ*g·m^−3^ recently revised by the Directive 2008/50/CE and proposed in the latest review of “Air Quality Guidelines” from WHO [15] as the reduction “target” level.

## 3. Results and Discussion

In this section, the estimated PM10 levels and health impact for both climate scenarios are analysed. The results obtained for short-term exposure (1 or 2 days), expressed as a number of attributable cases by all internal causes mortality, are presented and discussed. The increased number of attributable cases between the future and current pollution levels and the potential number of attributable cases prevented annually by reducing future PM10 concentrations to the legislation limit value (50 *μ*g·m^−3^) are also investigated.

### 3.1. Particulate Matter Levels under the IPCC SRES A2 Scenario

The simulated temperature increases under future climate almost reach 8.5°C over mid and southern Europe during the warm period of May–October [26]. These projections are in accordance to Rowell [50] who predicted that in winter the largest warming occurs over eastern Europe, up to 7°C, and in summer temperatures rise by 6–9°C south of about 50°N.

**Figure 7:**
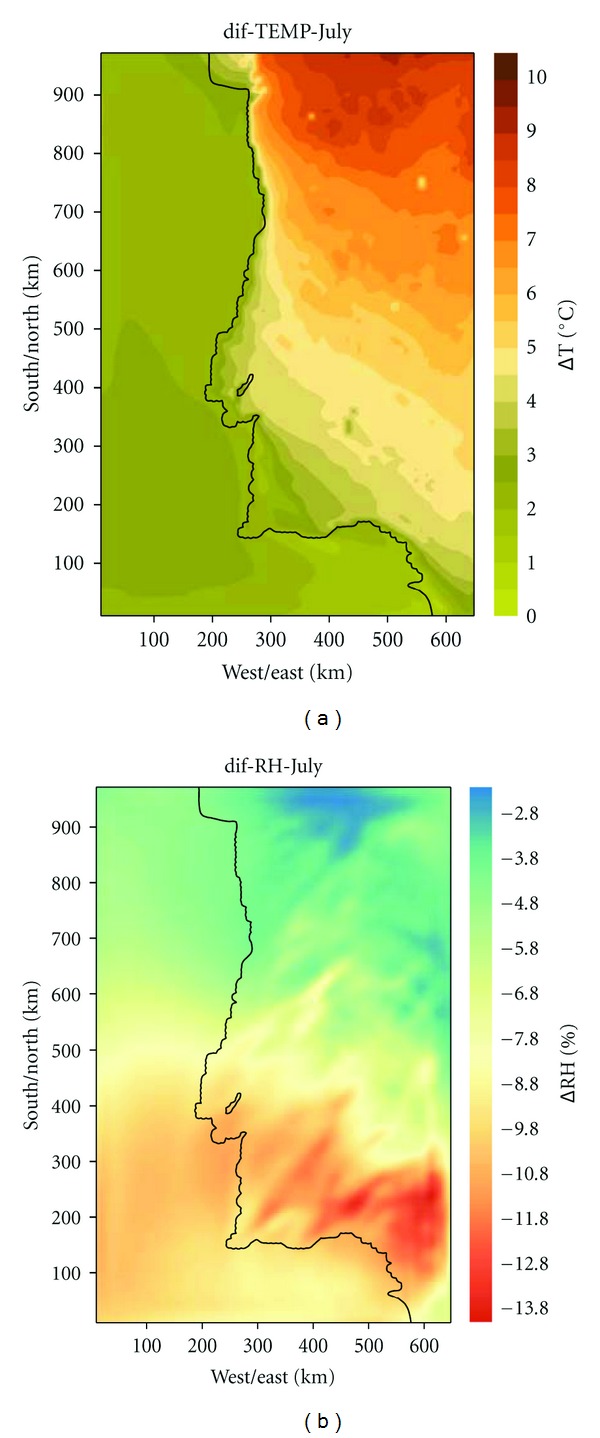
Temperature (°C) and relative humidity (%) differences between future and reference climates simulated with the MM5 model across Portugal for July.

In Figure 7, an example of the projected climatic changes over Portugal is presented for July showing the largest temperature increases over the northwestern part of Portugal reaching almost 10°C. Relative humidity (RH) will decrease significantly all over Portugal. The changes in the meteorological fields (temperature, RH, wind, boundary layer) will influence the pollutants dispersion and transformation in the atmosphere.

**Figure 8:**
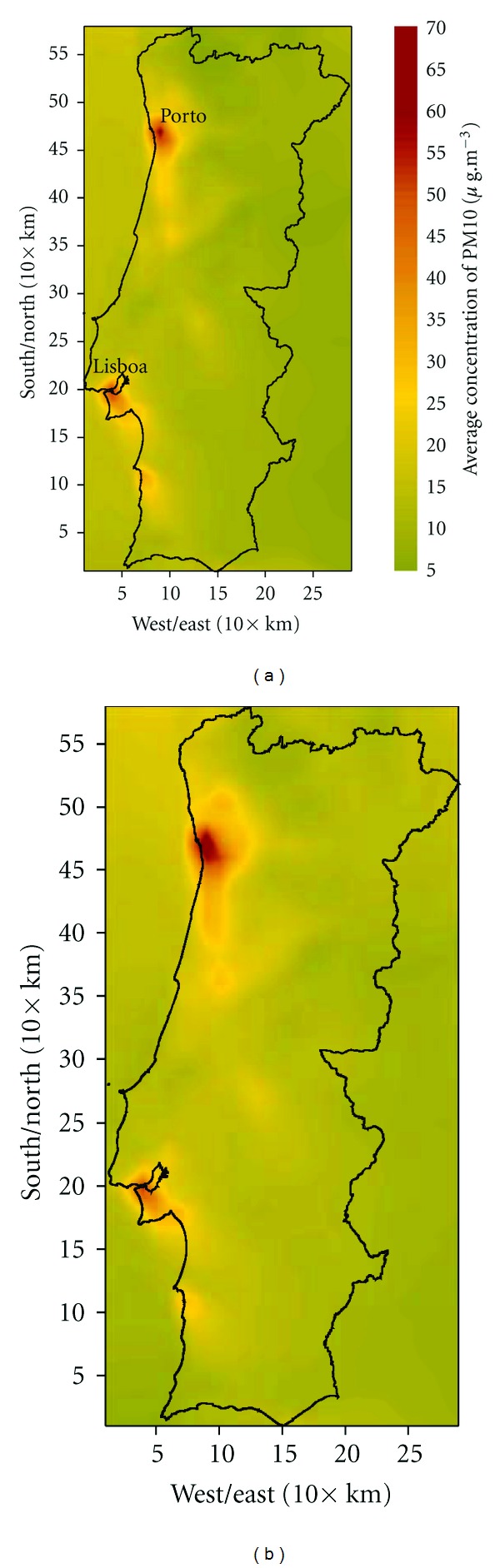
Average concentration of PM10 (*μ*g·m^−3^) for the simulated period (from May to October) for (a) current and (b) future climate scenario.

Wind speed, mixing height, and relative humidity are the meteorological variables believed to mostly influence PM concentrations. Stagnant conditions are thought to correlate with high PM concentrations, as they allow particulates to accumulate near the earth's surface. Although high wind speeds can increase ventilation, they are normally correlated with high PM concentrations because they allow the resuspension of particles from the ground, as well as long-range transport of particulates between regions. High PM concentrations are normally associated with dry conditions due to increased potential to resuspension of dust, soil, and other particles.  Figure 8 presents the average PM10 levels over Portugal over the simulation period for both climates based on hourly data provided by the air quality model.

For the overall simulation period, the maximum averaged PM10 levels increase from 60 *μ*g·m^−3^ to 72 *μ*g·m^−3^. In addition, over Porto and Lisbon regions, the area affected by higher concentrations also increases in future climate (Figure 8).

**Figure 9:**
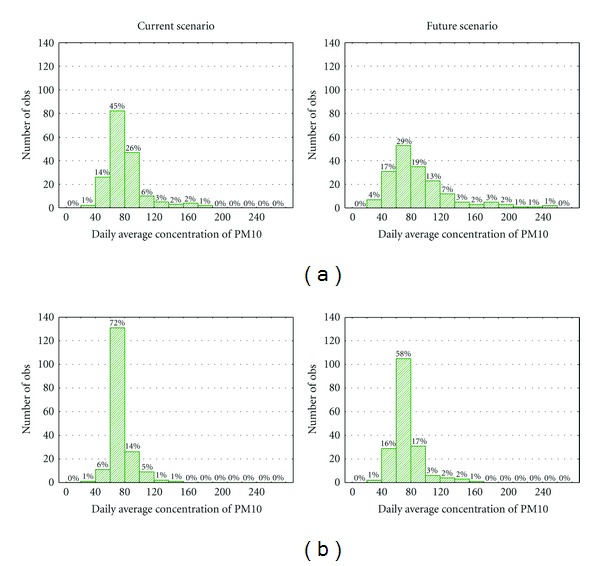
Frequency distribution of the PM10 concentrations for both climatic scenarios over the regions of (a) Porto and (b) Lisbon.

Additionally to the changes in the average pollution levels, the frequency distribution of the PM10 concentrations is also very important for the human health studies. In Figure 9, an example for the most affected regions of Porto and Lisbon is presented providing information on the frequency of pollution episodes under the two climate scenarios.

The frequency distribution of the PM10 concentrations for both climatic scenarios emphasizes that Lisbon and Porto districts present an elevated number of days with high PM10 levels in comparison with the legislation limit value for the daily average PM10 concentration of 50 *μ*g·m^−3^ that cannot be exceeded more than 35 times per year. Moreover, the climate-driven effect on PM10 levels will be more noticeable in Porto district leading to significant increase in the number of days with high daily average concentration.

### 3.2. Prognosis of Health Impact: Future versus Current Pollution Levels

**Figure 10:**
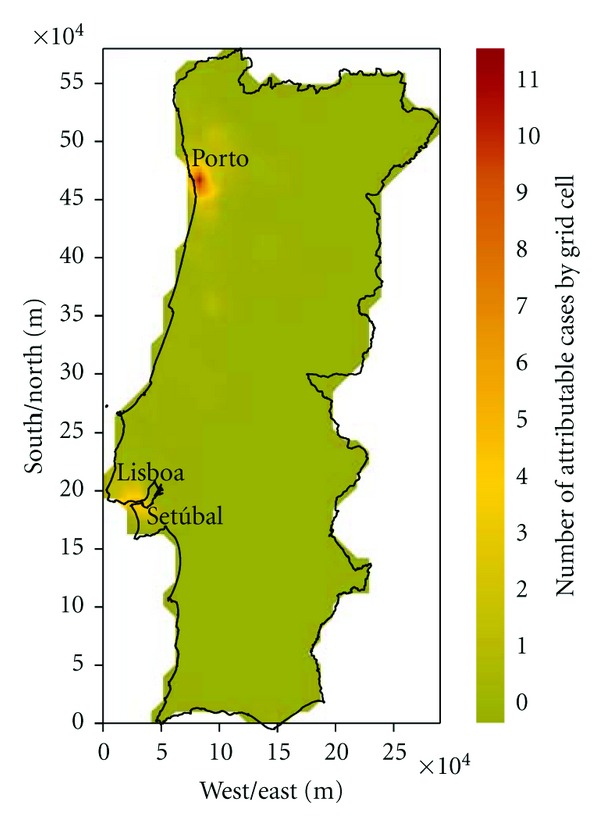
Spatial distribution of the increased number of attributable cases estimated by grid cell (10 × 10 km^2^) related to short-term PM10 exposure for future climate.

The health impact assessment based on the estimated changes in PM10 between the future and reference climate shows some locations with no significant increment in the number of attributable cases to short-term PM10 exposure while other locations show important increase in PM10-induced premature mortality (Figure 10). Since the number of estimated attributable cases depends on both air quality and the number of the inhabitants exposed, air quality changes in the densely populated areas of the country have a greater effect than air quality changes in less densely populated areas, in general. Modelling results suggest that worsened PM10 levels will coincide spatially with many of the most densely populated areas of the country (Figure 8).

As could be seen from Figure 10, the highest increase of the number of attributable cases under a future climate scenario would be expected in the Northern coastal region and Lisbon metropolitan area achieving a maximum augment of 11 cases by grid cell. The results presented in Table 1 highlight that the changes on the PM10 concentrations lead to a significant increase in the number of deaths in the future for most districts, especially those with the larger urban areas. additionally the Lisbon district is characterised by larger population size and the current mortality rate is higher, and the Porto district is the most affected (about 31% of total national deaths), reaching two times higher values than expected for the Lisbon district due to different prognosis of future pollution levels for these areas.

On the other hand, South of Portugal presents the lowest changes in the average mortality rate (Faro district: 0.9 (95% CI 0.6–1.2)) since the PM10 concentrations projected for 2100 will not increase significantly in comparison with the current pollution levels. At national level, about 203 (95% CI 137–271) more premature deaths per year are projected for 2100 in comparison to the current scenario due to indirect effect of climate change.

### 3.3. Prognosis of Health Impact: Future Pollution versus Legislation

**Figure 11:**
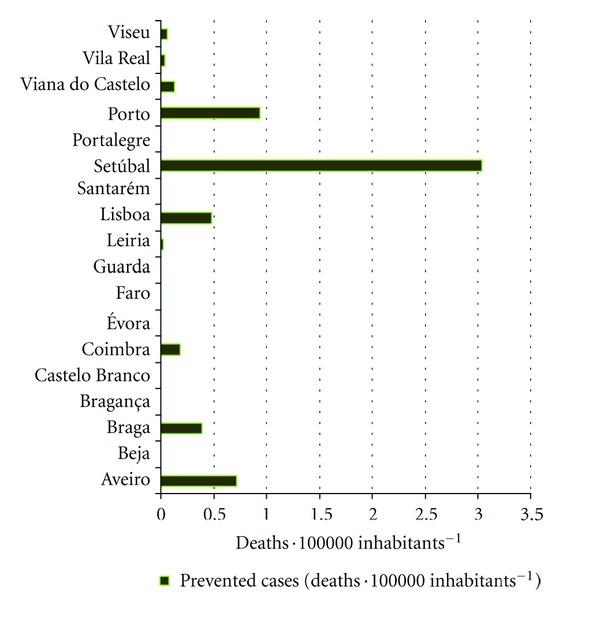
Prevented cases considering the fulfilment of the legislated value (deaths·100000 inhabitants^−1^).

Additionally to the impact assessment based on prognosis of future pollution, the benefit for human health related with potential reduction of PM10 to the legislation limit value (daily average concentration of 50 *μ*g·m^−3^) was analysed. The number of prevented cases for all internal causes mortality attributed to the short-term (1 or 2 days) exposure is quantified considering that no exceedances to the limit value will occur. The results for each district are presented in Figure 11.

Porto district will be the greatest benefited in case of the legislated value fulfilment that is possible to achieve with implementation of additional policy measures such as emission reductions. Therefore, if no air quality exceedances will occur, about 50 premature deaths related to PM10 exposure may be avoided annually, which corresponds to four times higher values than prevented cases estimated for the Lisbon district. As expected, this fact is related with highest increase in air pollution levels predicted for Porto in future climate.

**Figure 12:**
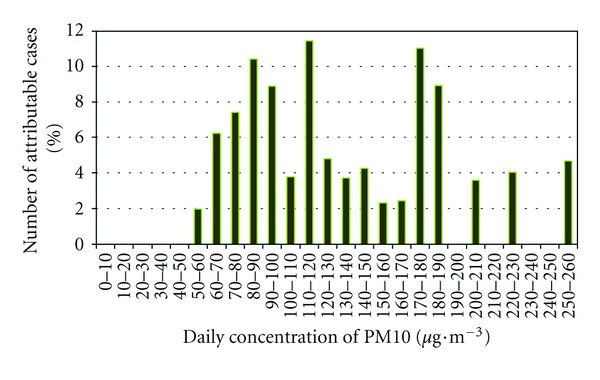
Distribution of the number of attributable cases (%) by PM10 concentration classes in Porto.

A more detailed analysis of the results obtained for the Porto area in terms of the number of attributable cases associated with different levels of exposure to PM10 is presented in Figure 12.

Although in Porto district average PM10 concentrations above 120 *μ*g·m^−3^ will occur in 13% of days, they are responsible for 50% of deaths attributable to air pollution. Thus emphasizing the greatest impact associated with “high pollution” days, despite their low frequency.

## 4. Conclusions

In this study, a quantitative assessment of the impact of climate change on human health related with short-term exposure to PM10 has been performed using combined atmospheric and impact assessment modelling. The modelling results obtained for the continental region of Portugal revealed that climate change alone will deeply impact the PM10 levels in the atmosphere. All the Portuguese districts will be negatively affected but negative effects on human health are more pronounced in major urban areas. The short-term variations in the PM10 concentration under future climate will potentially lead to an increase of 203 premature deaths per year in Portugal. The Porto district is the most affected in terms of occurrence of number of days with higher concentrations, consequently leading to the most significant increase in premature deaths that correspond to approximately 8% increase of its current mortality rate by all internal causes.

The pollution episodes with daily average PM10 concentration above the current legislated value (50 *μ*g·m^−3^) would be responsible for 81% of attributable cases. Although “high pollution” days have low frequency, they show the greatest impact and highlight the significant contribution of pollution peaks to acute exposure. Thus, the reduction of “high pollution” days with daily average concentration above 120 *μ*g·m^−3^ projected to the Porto district will avoid about 50% of premature deaths attributable to air pollution.

Although the hypothetical situation of what would happen if the predicted future climate conditions will occur in 2100 and assuming that PM10 precursor emissions and population maintain constant, the information provided in this study suggests that climate-driven changes on air pollutants and human health could be substantial. Therefore, additional efforts should be made to improve on this type of modelling approach in order to support local and wider-scale climate change mitigation and adaptation policies.

## Figures and Tables

**Table 1:** Increase of mortality attributable to PM10 pollution levels under the climate scenario in comparison with the reference situation. Values presented in parenthesis correspond to the 95% confidence interval (CI).

District	Mortality rate average and 95% CI (deaths per 100000 inhabitants)	Annual mortality average and 95% CI (deaths)
Aveiro	2.6 (1.7–3.5)	13 (9–18)
Beja	1.7 (1.1–2.2)	3 (2-3)
Braga	1.9 (1.3–2.6)	19 (12–25)
Bragança	2.0 (1.3−2.6)	3 (2–4)
Castelo Branco	1.7 (1.1–2.2)	3 (2–4)
Coimbra	2.5 (1.7–3.4)	11 (7–15)
Évora	1.4 (0.9–1.9)	3 (2-3)
Faro	0.9 (0.6–1.2)	3 (2–4)
Guarda	1.8 (1.2–2.5)	4 (3–5)
Leiria	1.6 (1.1–2.2)	8 (6–11)
Lisbon	1.3 (0.8–1.7)	26 (17–35)
Portalegre	1.8 (1.2–2.4)	2 (2-3)
Porto	3.7 (2.5−5.0)	62 (41–83)
Santarém	1.8 (1.2–2.3)	8 (5–11)
Setúbal	1.9 (1.2–2.5)	13 (9–18)
Viana do Castelo	2.4 (1.6–3.2)	8 (5–11)
Vila Real	1.9 (1.3–2.6)	6 (4–7)
Viseu	2.0 (1.3–2.6)	8 (5–11)

National	2.1 (1.4–2.8)	203 (135–271)
